# Managing Necrotizing Soft Tissue Infections of the Lower Limb: Microsurgical Reconstruction and Hospital Resource Demands—A Case Series from a Tertiary Referral Center

**DOI:** 10.3390/jcm14092997

**Published:** 2025-04-26

**Authors:** Georgios Karamitros, Michael P. Grant, Sharon Henry, Gregory A. Lamaris

**Affiliations:** 1 Division of Plastic and Reconstructive Surgery, R. Adams Cowley Shock Trauma Center, University of Maryland Medical Center, Baltimore, MD 21201, USA; gkaramitros@som.umaryland.edu (G.K.); michael.grant@som.umaryland.edu (M.P.G.); 2 Department of Surgery, R. Adams Cowley Shock Trauma Center, University of Maryland Medical Center, Baltimore, MD 21201, USA; sharon.henry@som.umaryland.edu

**Keywords:** necrotizing soft tissue infections, necrotizing fasciitis, lower extremity reconstruction, free tissue transfer, microsurgical reconstruction, anterolateral thigh flap, latissimus dorsi flap, surgical debridement, limb salvage, soft tissue defects, local flaps, dangling protocol, postoperative rehabilitation, regional anesthesia, pain management, multimodal analgesia, hospital length of stay, surgical outcomes, microsurgical limb reconstruction, wound healing, infection control, surgical resource utilization, retrospective case series

## Abstract

**Background:** Necrotizing soft tissue infections (NSTIs) of the lower extremities represent a surgical emergency with high morbidity, complex reconstruction, and considerable healthcare demands. Free tissue transfer (FTT) is increasingly utilized for limb salvage in extensive soft tissue defects, yet its implications for hospital resource utilization remain unclear. This study aims to compare clinical outcomes and perioperative resource demands between FTT and local flap (LF) reconstruction in NSTI patients. **Methods:** A retrospective case series was conducted at a tertiary referral center between September 2022 and January 2025, including eight patients with NSTI of the lower extremity (FTT, *n* = 4; LF, *n* = 4). Demographic data, comorbidities, surgical timing, complication profiles, and resource utilization metrics—including operative duration, hospitalization length, and number of procedures—were analyzed. All FTT cases underwent preoperative CT angiography as part of institutional protocol. **Results:** Mean time to definitive reconstruction was longer in the FTT group (17.25 vs. 8 days, *p* = 0.15), reflecting staged infection control. FTT procedures demonstrated significantly longer operative times (331.75 vs. 170.25 minutes, *p* = 0.015), but there was no significant difference in total hospital stay (34.75 vs. 27.71 days, *p* = 0.65). No cases of flap loss or venous congestion were observed, and outcomes were optimized via delayed dangling protocols. **Conclusions**: FTT is a viable and effective reconstructive modality for lower extremity NSTIs. Despite increased surgical complexity, FTT did not significantly increase hospital resource utilization, supporting its role in limb preservation among appropriately selected patients.

## 1. Introduction

Necrotizing soft tissue infections (NSTIs) represent a spectrum of life-threatening conditions affecting the skin and soft tissues, necessitating prompt recognition and urgent medical and surgical intervention [1,2,3,4,5]. While NSTIs can involve any anatomical region, the lower extremities are most frequently affected [4,6,7]. These infections can arise in both immunocompromised and otherwise healthy individuals, often secondary to trauma, prior surgical procedures, or even occur spontaneously. Necrotizing fasciitis (NF), a severe subset of NSTI, demonstrates an estimated incidence ranging from 0.4 to 32.64 cases per 100,000 population [2,8,9,10,11], with diabetes mellitus implicated in approximately one-third of cases [11,12,13,14]. Notably, 38–54% of NF cases involve the lower extremities [11,12], posing significant reconstructive challenges following surgical debridement, given the relative paucity of local tissues available for soft tissue coverage.

The microbial etiology of NSTI is categorized into three distinct types [15,16]. Type I infections are polymicrobial, involving aerobic and anaerobic bacteria, while Type II is characterized by monomicrobial infection, predominantly Streptococcus pyogenes and Staphylococcus aureus, with a more aggressive clinical course. Type III, albeit rare, is caused by Vibrio species, particularly in marine environments, often leading to fulminant sepsis [15,16]. Despite aggressive treatment, NSTIs exhibit high morbidity and mortality rates, with reported fatality rates reaching 33% [17,18,19]. Furthermore, amputation is required in up to 23.5% of cases when surgical debridement is delayed or inadequate [2,20,21,22,23,24,25,26,27].

Following debridement, lower extremity reconstruction is particularly challenging due to the frequent exposure of vital structures such as tendons, bones, and neurovascular elements. Conventional reconstructive approaches, including skin grafting, are often futile in cases with compromised vascularity, leading to poor healing outcomes [28]. Free tissue transfer (FTT) has emerged as a cornerstone of limb salvage in such scenarios, facilitating single-stage reconstruction, reducing hospital length of stay, and optimizing functional rehabilitation [29,30,31,32,33,34]. The anterolateral thigh (ALT) flap and other fasciocutaneous, musculocutaneous, or osteocutaneous flaps provide robust vascularity, minimal donor site morbidity, and versatile coverage options [35,36].

Despite its advantages, FTT in NSTI patients is fraught with challenges. The optimal timing of reconstruction remains debated—early flap placement may risk persistent infection, while delayed reconstruction prolongs wound healing and functional impairment. Additionally, patients with NSTIs frequently present with systemic inflammatory response syndrome (SIRS), hemodynamic instability, and multiple comorbidities, all of which increase surgical risk [7,37,38]. The necessity of harvesting a donor site in critically ill patients further complicates decision-making and requires a judicious surgical approach [39,40,41,42].

This study aims to evaluate the safety and efficacy of FTT in lower extremity NSTI reconstruction, particularly in medically complex patients. We present our institutional experience comparing outcomes between FTT and local flap (LF) reconstructions, with an emphasis on clinical outcomes, and healthcare resource utilization. Specifically, we assess whether FTT contributes to improved limb salvage without exacerbating morbidity or significantly prolonging hospital length of stay.

## 2. Hypothesis

This study investigates the following hypotheses: free tissue transfer (FTT) can be effectively utilized for the reconstruction of necrotizing fasciitis (NF) wounds of the lower extremity without leading to significant complications, such as flap failure or the need for additional reoperations. Furthermore, FTT can provide a reliable and safe reconstructive option for complex post-debridement wounds in NF cases without markedly increasing resource utilization, including hospital length of stay or the number of required surgical interventions.

While local flap reconstruction is typically reserved for cases with more limited soft tissue loss, and free tissue transfer is indicated in more extensive defects, we included both groups to reflect the full clinical spectrum of NSTI-related lower extremity reconstruction. This allowed for an internal reference point on surgical complexity, resource use, and postoperative course, recognizing that these groups differ in baseline severity and reconstructive indication. Our intention is not to equate these techniques, but to provide comparative context for institutional planning and outcomes evaluation.

## 3. Methods

### 3.1. Patient Selection

Following Institutional Review Board (IRB) exempt review (HP-00113288) at the University of Maryland, a retrospective review of health records was conducted to identify cases of lower limb necrotizing fasciitis (NF). Case identification was performed using the International Classification of Diseases (ICD)-10-CM codes for necrotizing fasciitis (M72.6) and myositis (M60). All identified cases underwent a comprehensive chart review to confirm the diagnosis of NF, ensuring that necrotic fascia was documented intraoperatively by the attending surgeon. To validate cases requiring free tissue transfer (FTT), patient records were cross-referenced with our institutional clinical registry of all FTT procedures, thereby ensuring data integrity and completeness.

This study was conducted at the R. Adams Cowley Shock Trauma Center and included cases managed between September 2022 and January 2025, in collaboration with the Divisions of Plastic Surgery and Soft Tissue Surgery within the Department of Surgery. The multidisciplinary nature of this approach provided a robust dataset for analysis.

The following de-identified patient variables were recorded for analysis: demographic data, including age and sex; surgical data, including type and size of the flap used, number of surgeries prior to flap surgery, duration of flap surgery, number of flap-related complications, number of flap failures, and hospital length of stay. NF-specific data were also collected, including anatomical site(s) affected, precipitating injury type, suspected causative infectious organism(s) based on culture results, and associated medical comorbidities. The comorbidities examined included age ≥ 50 years, smoking history, diabetes mellitus, hypertension and/or dyslipidemia (defined as requiring lipid-lowering therapy), and morbid obesity (BMI ≥ 35 kg/m^2^). Arterial perfusion to the lower extremities was initially assessed through physical examination, including palpation of distal pulses and evaluation of capillary refill. At our institution, all patients undergoing free tissue reconstruction for lower extremity defects undergo preoperative computed tomography angiography (CTA) as part of their standard vascular workup to evaluate arterial patency and map perforator anatomy. In cases where CTA raises concerns regarding aberrant anatomy or compromised inflow, formal catheter-based angiography is performed to further delineate vascular anatomy and guide surgical planning.

### 3.2. Data Analysis

Patient data were systematically compiled using Microsoft Excel, and statistical analysis was performed using STATA 17.0. Descriptive statistics were calculated for all variables. Normality was assessed using the Shapiro–Wilk test. Continuous variables were compared using Student’s *t*-test (normally distributed) or the Wilcoxon rank-sum test (non-normal distributions). Categorical variables were analyzed using Fisher’s exact test. A *p*-value < 0.05 was considered statistically significant.

## 4. Results

A total of eight patients with necrotizing fasciitis (NF) of the lower extremities that were admitted to our center required soft tissue coverage with vascularized tissue. Patients who underwent free tissue transfer (FTT) (*n* = 4; 1 female, 3 male) were compared with those who were hospitalized for NF but only required a local flap (LF) as part of their reconstruction (*n* = 4; 2 females, 2 male) (Table 1 and Table 2). The etiologies of NF included penetrating trauma (*n* = 2), surgical site infection (*n* = 1), blunt trauma (*n* = 1), pre-existing skin conditions (*n* = 2), and pressure ulcers secondary to paraplegia (*n* = 2). Five patients underwent duplex ultrasonography to assess lower extremity perfusion, and three of these underwent further evaluation with CT angiography. One patient was found to have clinically significant arterial obstruction, which was managed conservatively without need for endovascular or open revascularization.

Group A *β*-hemolytic *Streptococcus pyogenes* (GABS) was identified in the wound cultures of one patient in each cohort. In the LF group, wound cultures were positive in three of four patients, revealing a broader polymicrobial burden. Isolated pathogens included *Streptococcus dysgalactiae*, *Eggerthia catenaformis*, *Streptococcus anginosus*, *Arcanobacterium haemolyticum*, *Schaalia radingae*, and *Pseudomonas aeruginosa*. A comparative summary of the identified microorganisms across cohorts is provided in Table 3, highlighting a higher microbial diversity in the LF group, which may have implications for reconstructive planning and infection control strategies. Among the eight patients included in this case series, three (37.5%) had a documented diagnosis of diabetes mellitus—two of whom underwent local flap reconstruction and one who received a free tissue transfer. The most common comorbidities across the cohort were arterial hypertension (*n* = 5), obesity (*n* = 4), diabetes mellitus (*n* = 3), and chronic kidney disease (*n* = 2). In the local flap group (*n* = 4), comorbid conditions included arterial hypertension in three patients, diabetes mellitus in two, obesity in three, and chronic kidney disease in one. In the free flap group (*n* = 4), arterial hypertension was observed in two patients, diabetes mellitus in one, obesity in one, and chronic kidney disease in one. These comorbidities—well-established risk factors for necrotizing soft tissue infections (NSTIs)—may have influenced both the severity at presentation and the postoperative recovery trajectory. However, due to the limited sample size, formal subgroup analysis was not statistically feasible, and, as such, comparisons between groups should be interpreted with caution.

All patients received empiric broad-spectrum intravenous antibiotics upon diagnosis of necrotizing soft tissue infection, in accordance with institutional infectious disease protocols. The initial empiric regimens typically included a combination of vancomycin, piperacillin-tazobactam, and clindamycin. Antibiotic therapy was subsequently tailored based on wound culture sensitivities and infectious disease consultation. For patients with culture-confirmed monomicrobial *Streptococcus pyogenes* or other streptococcal species, penicillin derivatives were administered when appropriate. In polymicrobial infections, regimens were adjusted to cover anaerobic and Gram-negative organisms as indicated, with agents such as meropenem or cefepime used in select cases. Antibiotic treatment was maintained for a duration determined by infection severity, culture results, and clinical response, typically ranging from 10 to 21 days. Prophylactic antibiotics were also administered perioperatively during reconstructive procedures to minimize surgical site infection risk.

**Figure 1:**
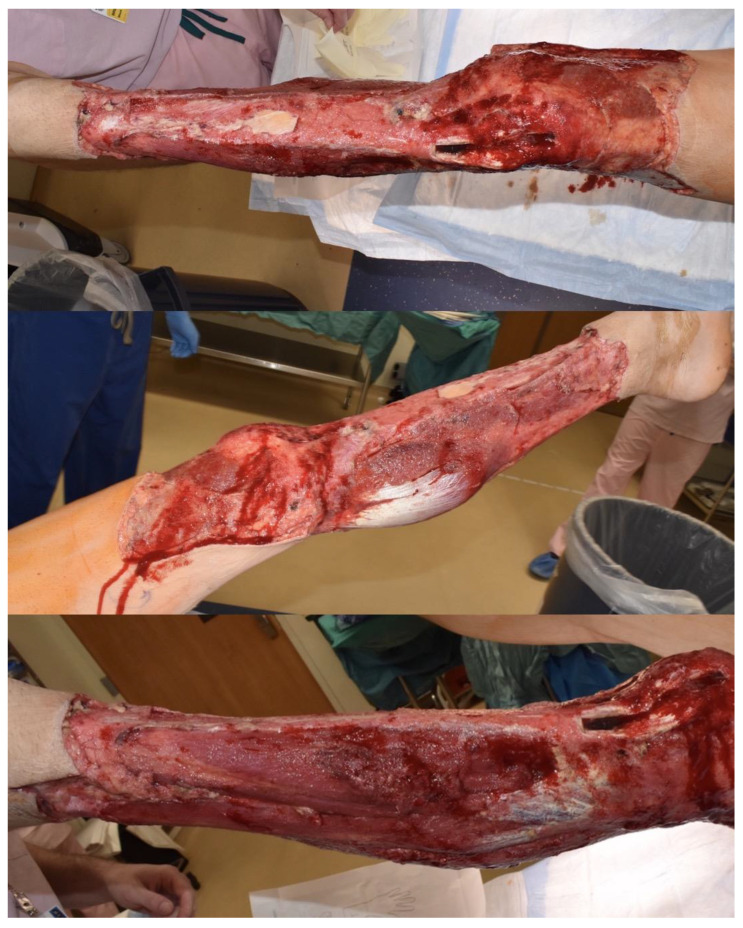
A 35 y.o. M developed necrotizing soft tissue infection of the left lower extremity following an elective endoscopic repair of torn anterior cruciate ligament of the knee. After multiple debridement, he had a circumferential wound spanning from the distal thigh to the ankle with exposed critical structures necessitating soft tissue reconstruction.

**Figure 2:**
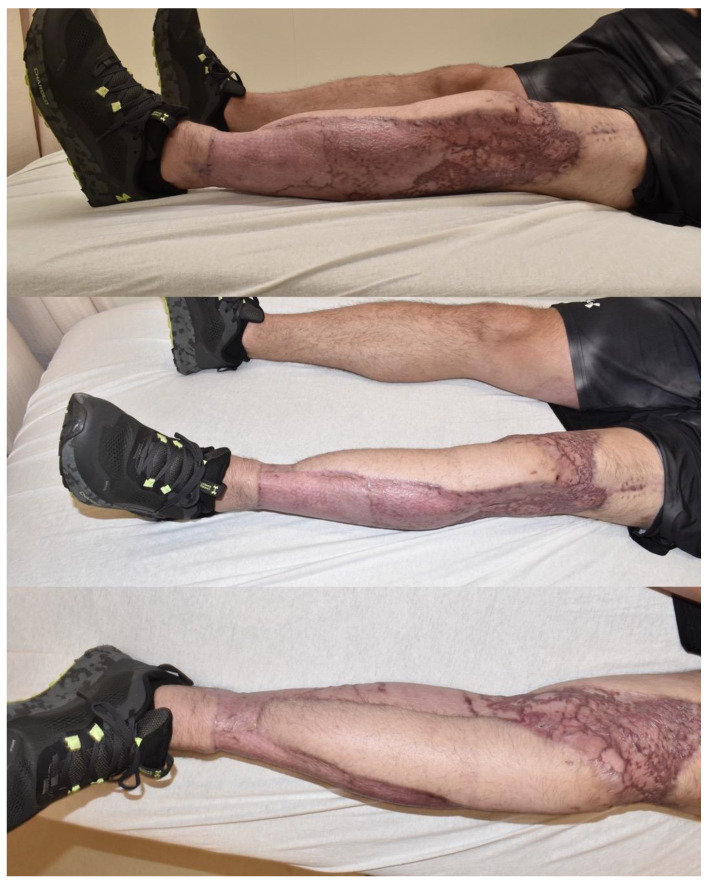
Post-operative appearance of wound 8 months following reconstruction. A musculocutaneous anterolateral thigh (ALT) flap was used (36 × 11 cm skin island and 16 × 6 cm vastus lateralis muscle) to cover the exposed bony structures of the knee and leg, while the posterior aspect of the leg was covered with a split thickness skin graft. At the time of the last follow-up (8 months), the patient had already returned to playing sports and was able to resume all his regular daily activities.

The timing of the flap procedure was determined based on patient stability, infection control, and minimization of necrotic tissue. Among the four flaps performed, three were chimeric or musculocutaneous anterolateral thigh (ALT) flaps incorporating the vastus lateralis (VL) muscle, while one was a latissimus dorsi (LD) flap utilized for definitive reconstruction (Table 1). Patient 3 (ID:3), a 35-year-old male with a surgical site infection, underwent reconstruction with a chimeric ALT-VL flap (muscle flap size: 16 × 6 cm, skin island size: 36 × 11 cm) after extensive debridement of the left lower extremity (Figure 1). Postoperatively, the patient achieved complete flap integration with no complications (Figure 2). No complications were observed at the FTT recipient sites. However, donor site complications in the FTT cohort included hematoma (*n* = 1) and infection (*n* = 1). In contrast, the only complication in the LF cohort was wound dehiscence (*n* = 1), which was managed conservatively with local wound care and antibiotics.

Comparative analysis revealed no statistically significant difference in the mean age between the two cohorts (*p* = 0.78). The only statistically significant difference between groups was the duration of surgery, which was longer in the FTT cohort (*p* < 0.05). No significant difference was found in total hospital length of stay between the FTT and LF groups (34.75 vs. 27.71 days, *p* = 0.65) (Table 4).

FTT procedures were performed at a mean of 17.25 days post admission, whereas definitive surgery in the LF group occurred at a mean of 8 days post admission (*p* = 0.15) (Table 4). Patients in the FTT group underwent a mean of 4.25 procedures prior to free tissue transfer, compared to a mean of 2.5 procedures in the LF group before definitive local flap coverage. Finally, no significant difference was observed between cohorts in the total number of surgeries/debridements required to achieve disease control (7.75 vs. 5.5, *p* = 0.63) (Table 4).

## 5. Discussion

The initial management of necrotizing fasciitis (NF) necessitates multiple debridements, potentially leading to significant soft tissue loss and, in severe cases, limb amputation [18]. Achieving stable wound coverage is essential for optimizing functional recovery and reducing the risk of secondary infections. Free tissue transfer (FTT), a cornerstone of reconstructive surgery for nearly four decades [43,44], was initially reserved for select cases when traditional reconstructive methods were insufficient [35]. However, with advancements in microsurgery, FTT has become a preferred approach, demonstrating success rates approaching 100% in specialized centers [45,46]. In our cohort, we successfully employed microvascular FTT for NF reconstruction without encountering any flap losses. For instance, Patient 3 (ID:3) demonstrated robust soft tissue coverage and functional recovery after ALT-VL flap reconstruction (Figure 2), underscoring the utility of FTT in complex defects.

A key advancement in lower limb salvage is the emphasis on early free flap reconstruction (FFR). The timing of FTT plays a crucial role in optimizing outcomes. Studies suggest that early intervention (within 24–48 h) in lower extremity injuries reduces hospital stays, lowers infection rates [47,48], and improves functional outcomes [49]. However, in the case of NSTI, successful reconstruction hinges more on adequate infection control prior to FTT to mitigate the risk of flap failure. Our findings indicate that the paradigm of early soft tissue coverage following lower extremity trauma does not hold in the context of NF, as the mean time from admission to flap surgery was 17.25 (±7.63) days in the FTT group and 8 (±6.04) days in the local flap cohort. This delay in NF-related reconstruction contrasts with the 48 h window often deemed optimal for lower limb salvage in trauma cases [48], but holds relevance with other case series from other centers investigating the outcomes of FTT in NF management [4]. Although no statistically significant differences were observed in preoperative hospitalization duration (*p* = 0.15), a trend toward prolonged hospitalization was noted in the FTT cohort (17.25 vs. 8 days). This likely reflects the need for systemic optimization and multiple operative debridements to achieve adequate infection control prior to definitive microsurgical reconstruction. In contrast, patients who underwent local flap (LF) procedures likely had less extensive disease, resulting in smaller soft tissue defects that permitted earlier intervention and shorter preoperative hospitalization. This trend is further supported by the difference in the mean number of debridement procedures required before flap coverage, with LF-treated cases requiring fewer debridements compared to the FTT cohort (2.5 vs. 4.25 surgeries). These findings underscore the critical role of patient selection and surgical timing in NF reconstruction, emphasizing that FTT is often reserved for more complex cases where additional time is required for infection control and wound bed optimization.

When evaluating hospital resource utilization, including total length of stay and number of surgical interventions, our findings suggest that FTT can be safely used for NF wound coverage without significantly increasing hospital resource consumption. However, this study has limited statistical power due to the small sample size. Despite the high volume of NSTI cases at our institution, NF of the lower extremities remains rare, limiting generalizability. This introduces the potential for type II error, wherein a true difference in outcomes may not have been detected [50]. While our study did not achieve statistical significance, we observed a clinically meaningful trend toward shorter hospital duration in the local flap cohort (34.75 vs. 27.71 days, *p* = 0.65). These findings warrant further investigation through larger, multicenter prospective studies to refine evidence-based reconstructive strategies in NF management. Furthermore, while our study compared reconstructive options, future investigations should also include amputation (e.g., below-knee amputation) as a comparator to comprehensively evaluate resource utilization and functional outcomes.

Donor site complications were observed in two FFT patients: one with a superficial wound infection and one with a hematoma. These were managed conservatively without surgical reintervention and had a limited impact on resource utilization. In the LF cohort, one patient developed wound dehiscence, which was similarly managed with local wound care and pressure relief, also without additional operative intervention. While these complications increased the need for focused postoperative management, they did not significantly alter the overall hospitalization course or resource burden in either group.

Postoperative infection control remains a critical factor in reconstructive success. Initial broad-spectrum antibiotic coverage, followed by targeted therapy based on culture results, is widely accepted [15,16]. Interestingly, only one patient in the FTT group had identified wound pathogens (*Streptococcus pyogenes* and *Escherichia coli*), whereas three patients in the LF group had culture-positive wound infections. The microorganisms identified in the LF group included *Streptococcus dysgalactiae*, *Eggerthia catenaformis*, *Streptococcus anginosus*, *Arcanobacterium haemolyticum*, and *Schaalia radingae*—notable findings given that some of these pathogens have not been widely reported in NF cases [51]. Additionally, one case involved multidrug-resistant *Pseudomonas aeruginosa*, highlighting the diverse microbial spectrum associated with NF wounds [52,53].

Another critical rehabilitation strategy in NF-related free tissue transfer (FTT) is the implementation of a progressive venous pressure exposure protocol, commonly referred to as the dangling protocol, to optimize venous drainage and prevent venous congestion—key factors for flap survival [54,55,56,57,58,59,60,61,62,63]. At our institution, strict extremity elevation is enforced immediately postoperatively, with a gradual initiation of the dangling protocol beginning on postoperative day 10. The duration of exposure is progressively increased over four weeks until full weight bearing is achieved without restriction.

There is ongoing debate regarding the optimal timing of venous pressure exposure, with proponents of both “early” and “delayed” dangling protocols [59,64,65,66,67]. However, current protocols primarily focus on microvascular FTT in lower extremity reconstruction without considering the etiology of the underlying defect. Given the history of necrotic tissue debridement and the potential for residual bacterial load in NF cases, we adopted a more conservative approach, prioritizing optimal flap integration before subjecting the limb to venous pressure changes. This delayed dangling strategy was aimed at mitigating the risk of venous congestion and ensuring maximal flap take. Our protocol was highly effective, with no cases of venous congestion or flap loss observed. In addition, early range of motion (ROM) exercises were initiated to preserve joint mobility and prevent long-term functional deficits, particularly in cases with bone or tendon exposure. This approach underscores the need for condition-specific rehabilitation protocols in microsurgical limb reconstruction, particularly in the context of infection-driven defects such as NF.

There is substantial evidence highlighting the adverse effects of opioids and their potential for long-term dependence [68]. Given the need for effective pain management, particularly during frequent and painful dressing changes in this patient population, we implemented a multimodal analgesia strategy centered around regional anesthesia with continuous nerve block catheters (e.g., femoral or sciatic nerve blocks) to provide prolonged postoperative pain control. This catheter-based approach offers sustained analgesia, significantly reducing opioid consumption and its associated adverse effects while facilitating early mobilization. Compared to epidural anesthesia, regional techniques are less invasive, have a lower risk of complications, and provide targeted pain relief with superior patient tolerance. While well-established across various surgical disciplines [69,70,71,72], the application of regional anesthesia in the postoperative management of NSTI reconstruction remains relatively novel. At our institution, this protocol has become the standard of care for NF cases, proving particularly advantageous in mitigating procedural pain and enhancing overall patient comfort and recovery.

Beyond pain management, optimizing functional and aesthetic outcomes following FTT often necessitates secondary procedures, including flap contouring, debulking, and orthopedic interventions such as manipulation under anesthesia (MUA) for joint stiffness or bone exposure [73,74,75]. However, in our cohort, no additional surgeries were required to refine limb function or aesthetics. This may be attributed to the anatomical distribution of NF cases, as only one patient required ankle reconstruction, a region where bulk reduction is often necessary for proper footwear accommodation. In this instance, an anterolateral thigh (ALT) flap was employed, incorporating a fasciocutaneous component with a segment of vastus lateralis (VL) muscle to provide robust bone coverage while minimizing excess volume, thereby eliminating the need for secondary revisions. These findings underscore the importance of precise intraoperative planning in achieving optimal reconstructive outcomes while reducing the burden of additional interventions, further reinforcing the utility of microsurgical reconstruction in complex NF cases.

Lastly, there remains limited data on the influence of comorbidities on FTT outcomes in NF or whether FTT necessitates additional healthcare resources. Gawaziuk et al. conducted the largest case series on FTT in NF patients, analyzing individuals who underwent FTT over a six-year period [4]. Our study corroborates their findings, demonstrating comparable comorbidities and resource utilization between FTT and LF patients, with universally successful outcomes and minimal complications. NF patients frequently present with diabetes mellitus, obesity, and smoking history—factors known to increase flap failure risk [7]. Despite these challenges, our findings support the efficacy of FTT in achieving durable reconstruction and limb preservation when meticulous debridement and perioperative optimization are ensured.

## 6. Limitations

This study is subject to inherent limitations due to its retrospective case-series design and small sample size, which constrain statistical power and increase the risk of type II error, potentially obscuring true differences between groups [50,76,77]. While NF of the lower extremity requiring FTT is a rare clinical entity, limiting large-scale study feasibility, our findings remain clinically meaningful given the complexity and acuity of this patient population. Although comorbidities such as diabetes mellitus, arterial hypertension, obesity, and chronic kidney disease were documented and descriptively reported, the limited cohort size precluded formal subgroup analyses to determine their specific influence on reconstructive strategy or outcomes. Epidemiologically, such analyses in small samples risk generating spurious or underpowered associations due to inflated variance and model overfitting. Therefore, while these conditions likely influenced initial presentation and healing trajectories, the study design does not allow for definitive causal inferences regarding their impact.

Additionally, the lack of statistical significance in some comparisons should not discount observed clinical trends, which may become significant in larger, hypothesis-driven cohorts. Despite these constraints, our institution’s sustained clinical experience in managing complex NF cases contributes to the internal validity and translational value of the findings. Future multicenter prospective studies with larger sample sizes and standardized reporting of comorbid conditions are warranted to refine patient selection criteria, reconstructive algorithms, and outcome benchmarking.

This study assesses clinical resource utilization based on surrogate endpoints, including operative time, number of surgical interventions, and total hospital length of stay. While informative, these metrics do not constitute a formal economic analysis. A cost-effectiveness evaluation would require access to detailed direct and indirect cost data, the valuation of clinical outcomes, and modeling across relevant time horizons [78]—all of which fall beyond the scope of this retrospective case series. Future research employing prospective designs, economic datasets, or health economic modeling frameworks will be essential to further elucidate the financial implications of complex reconstructive strategies in NSTI management.

## 7. Conclusions

The utilization of microsurgical free tissue transfer has significantly advanced the management of lower extremity reconstruction following necrotizing soft tissue infections (NSTI). At the R. Adams Cowley Shock Trauma Center, our multidisciplinary team has gained valuable experience in reconstructing NF of the lower extremity, focusing on optimal flap selection, infection control, pain management, and rehabilitation. These efforts have led to improved functional outcomes, limb preservation, and patient satisfaction. Our findings suggest that FTT is a viable reconstructive option for NF patients without significantly increasing resource utilization. Further studies with larger sample sizes are necessary to validate these findings and refine best practices for NF-related limb salvage.

## Figures and Tables

**Table 1:** Free Flap/Tissue Transfer (FFT) Case Series.

ID	Sex	Age	History	Location	Organism	Days to Flap from First Debridement	FTT Type	Muscle Flap Size, cm × cm (cm^2^)	Skin Island Size, cm × cm (cm^2^)	Complications	Surgery Time (min)	Ischemia Time (min)
1	Male	27	Pyoderma gangrenosum ulcer	L lower leg/knee	Not identified ^1^	19	Chimeric ALT-VL	Not specified	22 × 11 (242)	Infection of donor site	330	Not specified
2	Male	34	Penetrating trauma	L foot/ankle	GABS ^2^ and E.coli	19	Chimeric ALT-VL	18 × 8 (144)	11 × 24 (264)	None	240	54
3	Male	35	Surgical site infection	L lower leg	Not identified	5	Chimeric ALT-VL	16 × 6 (96)	36 × 11 (396)	None	375	Not specified
4	Female	65	Blunt trauma	L lower leg	Not identified	26	LD	38 × 23 (874)	-	Heamatoma (donor site)	382	65

^1^ Not identified: indicates cultures that had no growth. ^2^ Group A beta-hemolytic Streptococcus species.

**Table 2:** Local Flap(LF) Case Series.

ID	Sex	Age	History	Location	Organism	Days to Flap from First Debridement	LF Type	Defect Size, cm × cm (cm^2^)	Complications	Surgery Time (min)
1	Male	37	T16 paraplegia Girldstone procedure	L lower leg (trochanter and ischium)	Not identified	7	VL-RF	10 × 14 (140)	None	255
2	Female	60	Pain and redness	L lower leg (upper thigh, groin, perineum)	Eggerthia catenaformis, Streptococcus anginosus, Schaalia radingae	17	Sartorius-RF	35 × 55 (1925)	None	126
3	Female	30	Penetrating injury, abscess formation	L lower leg (groin)	Streptococcus dysgalactiae, Arcanobacterium haemoliticum	8	RF	3 × 8 (24)	None	102
4	Male	43	Bilateral paraplegia	L lower leg (Ischial tuberosity)	Pseudomonas aeroginosa ^3^	0	Biceps femoris	Not specified	Wound dehiscence	198

^3^ Multi-drug resistant, carbapenemase detected.

**Table 3:** Likely causative organisms in patients with NF of the lower extremities that underwent FTT compared to LF.

GABS ^4^	FTT (*n* = 4) 1 (25%)	LF (*n* = 4) 1 (25%) ^5^
*S. aureus*	0 (0%)	0 (0%)
*E. coli*	1 (25%)	0 (0%)
Other	0 (0%)	3 (75%)

^4^ GABS: Group A beta-hemolytic Streptococcus species. ^5^ In the LF group the GABS was a Streptococcus dysgalactiae.

**Table 4:** Demographic data of patients with NF of the lower extremities that underwent FFT and no FFT.

	FTT (*n* = 4)	LF (*n* = 4)	*p* Value
Age (±SD)	39.25 ± 15.59	42.5 ± 11.1	0.78
Sex (male)	3 (75%)	2 (50%)	0.47
Surgery duration (min ± SD)	331.75 ± 56.61	170.25 ± 60.35	0.015
Days of hospital stay (total)	34.75 ± 17.6	27.71 ± 18.71	0.65
Total number of operations	7.75 ± 5.36	5.5 ± 5.59	0.63
Days from admission to flap surgery	17.25 ± 7.63	8 ± 6.04	0.15
Surgeries before flap	4.25 ± 2.86	2.5 ± 2.29	0.44
Positive smoking status	1/4 (25%)	3/4 (75%)	0.16

## Data Availability

The data presented in this study are available in the article and tables. Raw data are not publicly available due to privacy and ethical restrictions imposed by the Institutional Review Board (HP-00113288, approved on 2025-02-03).
